# A Five-LLPS Gene Risk Score Prognostic Signature Predicts Survival in Hepatocellular Carcinoma

**DOI:** 10.1155/2023/7299276

**Published:** 2023-02-22

**Authors:** Wenwen Lai, Defu Li, Qiong Ge, Yehong Yan, Shiwen Luo, Quqin Lu

**Affiliations:** ^1^Jiangxi Provincial Key Laboratory of Preventive Medicine, Nanchang University, Nanchang, Jiangxi, China; ^2^Department of Biostatistics and Epidemiology, School of Public Health, Nanchang University, Nanchang, Jiangxi, China; ^3^Department of General Surgery, The First Affiliated Hospital of Nanchang University, Nanchang, Jiangxi, China; ^4^Center for Experimental Medicine, The First Affiliated Hospital of Nanchang University, Nanchang, Jiangxi, China

## Abstract

**Background:**

Primary liver cancer, dominated by hepatocellular carcinoma (HCC), is one of the most common cancer types and the third leading cause of cancer death in 2020. Previous studies have shown that liquid–liquid phase separation (LLPS) plays an important role in the occurrence and development of cancer including HCC, but its influence on the patient prognosis is still unknown. It is necessary to explore the effect of LLPS genes on prognosis to accurately forecast the prognosis of HCC patients and identify relevant targeted therapeutic sites.

**Methods:**

Using The Cancer Genome Atlas dataset and PhaSepDB dataset, we identified LLPS genes linked to the overall survival (OS) of HCC patients. We applied Least Absolute Shrinkage and Selection Operator (LASSO) Cox penalized regression analysis to choose the best genes for the risk score prognostic signature. We then analysed the validation dataset and evaluated the effectiveness of the risk score prognostic signature. Finally, we performed quantitative real-time PCR experiments to validate the genes in the prognostic signature.

**Results:**

We identified 43 differentially expressed LLPS genes that were associated with the OS of HCC patients. Five of these genes (*BMX*, *FYN*, *KPNA2*, *PFKFB4*, and *SPP1*) were selected to generate a prognostic risk score signature. Patients in the low-risk group were associated with better OS than those in the high-risk group in both the training dataset and the validation dataset. We found that *BMX* and *FYN* had lower expression levels in HCC tumour tissues, whereas *KPNA2*, *PFKFB4*, and *SPP1* had higher expression levels in HCC tumour tissues. The validation demonstrated that the five-LLPS gene risk score signature has the capability of predicting the OS of HCC patients.

**Conclusion:**

Our study constructed a five-LLPS gene risk score signature that can be applied as an effective and convenient prognostic tool. These five genes might serve as potential targets for therapy and the treatment of HCC.

## 1. Introduction

Primary liver cancer is one of the most common cancer types. In 2020, there were approximately 906,000 new cases and 830,000 deaths worldwide, making it the third leading cause of cancer death. Hepatocellular carcinoma (HCC) represents approximately 80% of all primary liver cancer cases [1]. Many factors contribute to the development and progression of HCC, including hepatitis B virus (HBV) [2], hepatitis C virus (HCV) [3], alcohol addiction, and metabolic liver disease [4]. Currently, the main treatments for HCC include radiotherapy, chemotherapy, surgical resection, and liver transplantation [5, 6]. Unfortunately, the prognosis of HCC patients remains poor, due to the high heterogeneity of HCC despite great advances in treatment over the past decades [7]. Conventional signatures utilizing clinical tumour-node-metastasis staging, vascular invasion, and other parameters aid the prediction of HCC prognosis [8]. However, in the era of precision medicine, it has been difficult to adopt personalized treatment with a single indicator or biomarker for HCC patients, since HCC is a multifactorial disease. Therefore, it is necessary to develop a multi-indicator tool to assist clinicians in optimizing treatments for HCC patients, thus improving their prognosis.

Eukaryotic cells coordinate numerous biochemical reactions spatially and temporally. Key to such coordination is functional compartmentalization of intracellular space. Compartmentalization can be achieved by intracellular membranes, which wrap around organelles and act as physical barriers [9]. However, recent reports have revealed that the majority of cellular processes are compartmentalized in biomolecular condensates formed by liquid–liquid phase separation (LLPS) [10, 11]. LLPS involves different densities, which are composed of intracellular biological macromolecules similar to a liquid drop, making contact and forming a relatively closed environment under the action of driving force to allow certain molecules in the cell to come together and perform a certain physiological function within the cell [12, 13]. Most research on LLPS are fairly new, but accumulating evidence suggests that it is closely linked to the occurrence and development of cancer. It can induce the occurrence and development of cancer in a variety of ways, such as transcription, cell signalling, and DNA repair [14]. Chen et al. reported that circVAMP3 negatively regulates the proliferation and metastasis of HCC cells in vitro and in vivo by driving phase separation of CAPRIN1 [15]. Gaglia et al. found that biomolecular condensates and LLPS of heat-shock factor 1, a transcriptional regulator of chaperones, affect the development of cancer [16]. Huang et al. showed that the guanine nucleotide exchange factor Son of Sevenless takes part in the occurrence and development of cancer through regulating RAS signalling [17]. Others demonstrated that LLPS of 53BP1 determines the behaviour of DNA repair compartments and that disrupting 53BP1 phase separation impairs 53BP1-dependent induction of p53 and diminishes p53 target gene expression [18]. The newly emerging principles of LLPS are expected to help people understand the life process of cells from a new perspective.

Previous studies have shown that LLPS plays an important role in the occurrence and development of HCC; however, its effect on the prognosis of HCC patients is still unknown. In this study, we aimed to investigate the prognostic role of the special mechanism in HCC patients and identify relevant targeted therapeutic sites; therefore, as to assist clinicians in personalized treatment for HCC patients to improve their prognosis. Finally, we developed a five-LLPS gene risk score prognostic signature to predict survival in HCC using the The Cancer Genome Atlas-liver hepatocellular carcinoma (TCGA-LIHC) dataset and PhaSepDB dataset.

## 2. Materials and Methods

### 2.1. The HCC Patient Dataset and PhaSepDB Dataset

**Figure 1:**
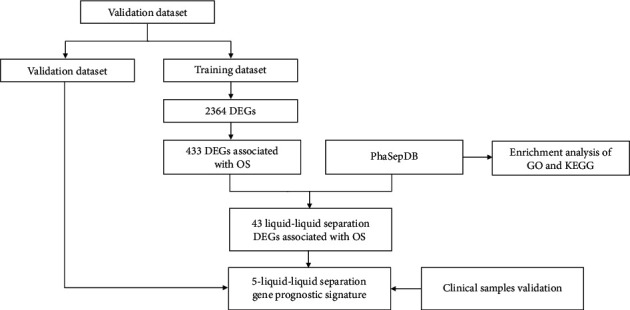
The process of constructing the five-LLPS gene risk score signature. First, 43 differentially expressed LLPS genes associated with OS in HCC patients were identified by differential expression analysis and univariate Cox proportional hazards regression analysis. Next, Least Absolute Shrinkage and Selection Operator (LASSO) Cox penalized regression analysis was applied to construct a gene risk score signature for prognosis prediction. Then, the gene risk score signature was generated based on five LLPS genes (*BMX*, *FYN*, *KPNA2*, *PFKFB4*, and *SPP1*). Finally, the five-LLPS gene risk score signature was validated by the validation dataset and clinical samples using experiments.

We obtained the RNA-seq data and clinical information of patients with HCC from the TCGA-LIHC dataset (https://cancergenome.nih.gov/). The following patient cases were excluded: first, cases with no follow-up time or survival status, and second, patients who had clinical information, but no corresponding RNA-seq data. A total of 343 HCC patients and 50 controls were enrolled in the study. The 343 tumour cohort were randomly divided into a training dataset (*n* = 240) and a validation dataset (*n* = 103) in a ratio of 7 to 3. The LLPS gene data were obtained from the PhaSepDB dataset (http://db.phasep.pro/). The workflow of the analysis conducted in this study was shown in Figure 1.

### 2.2. Enrichment Analysis of GO Functions and KEGG Pathways

Within the LLPS genes from the PhaSepDB dataset, Gene Ontology (GO) functions and Kyoto Encyclopedia of Genes and Genomes (KEGG) pathway analysis were conducted using WebGestalt (WebGestalt: WEB-based GEne SeT AnaLysis Toolkit, RRID:SCR_006786). A gene set with *P* < 0.05 and false discovery rate <0.05 was classified as significantly enriched.

### 2.3. Analysis of Differentially Expressed Genes Associated with Overall Survival in HCC Patients

We first performed log2 transformation for the expression profiles, which were then used for differentially expressed analyses with the “Limma” version 4.0.3 R package [19]. Genes with |log_2_FC| > 2 and *P* < 0.05 were categorized as differentially expressed genes (DEGs). We next performed univariate Cox proportional hazards regression analysis to assess the relationship between the DEG expression and the overall survival (OS) of HCC patients.

### 2.4. Prognostic Signature Construction

We performed the intersection of DEGs associated with the OS of HCC patients and LLPS genes from the PhaSepDB dataset to obtain the best LLPS genes for the prognostic signature. Next, we performed LASSO Cox penalized regression analysis using the R package “glmnet” [20], and we selected the genes with non-zero coefficients to build a risk score prognostic signature. We calculated the risk score for each HCC patient in the training dataset, based on the results of LASSO Cox penalized regression analysis. Then, we divided HCC patients into high- and low-risk groups based on the median risk score and performed Kaplan–Meier survival analysis.

### 2.5. Prognostic Signature Validation

After the risk score prognostic signature was generated, a dataset from the TCGA-LIHC dataset including 103 patients with complete OS information was used as the validation dataset. First, we performed time-dependent receiver operating characteristic (ROC) curve analysis for the 12-, 36-, and 60-month survival predictions using the R package “survivalROC” [21]. Subsequently, we divided HCC patients into high- and low-risk groups in the same way and conducted Kaplan–Meier survival analysis to analyse the association between the risk score prognostic signature and the OS of HCC patients. We determined the significance of differences in survival between the two groups with the log-rank test. All statistical analyses were performed using the R 4.0.3, and a two-sided *P* < 0.05 was considered statistically significant.

### 2.6. Clinical Samples Validation

To explore the biological functions and characteristics of the genes in the prognostic signature that we constructed, we performed quantitative real-time PCR (qRT-PCR) experiments to validate our results. We collected eight pairs of tumour tissue samples and their corresponding adjacent normal tissue samples of patients with HCC from the First Affiliated Hospital of Nanchang University, Jiangxi, China. All patients included in our study were definitively diagnosed with HCC and had not undergone radiotherapy or chemotherapy before surgical resection treatment. When a patient after surgical resection treatment, we obtained the fresh tumour tissue sample and corresponding adjacent normal tissue sample. The information of patients was shown in Table 1. Total RNA of clinical samples was extracted by using TRIzol® Reagent (Life Technologies™, USA) and 1 *μ*g total RNA was reversed to cDNA in 20 *μ*l reaction system with PrimeScript® RT reagent Kit with gDNA Eraser (Takara, Japan, DRR047A). qRT-PCR was performed by using SYBR® Premix Ex Taq™ Tli RnaseH Plus (Takara, Japan, DRR820A) with the QuantStudio™ 3 Real-Time PCR Instrument (Applied Biosystems, USA). The primer concentration used for the qRT-PCR is 10 *μ*M. Relative messenger ribonucleic acid (mRNA) expression levels of target genes were normalized to GAPDH using the 2^−△△CT^ algorithm. Every qRT-PCR experiment was performed in triplicate and repeated three times independently. The primers used were listed in Table 2. This study was approved by the Ethics Committee of the First Affiliated Hospital of Nanchang University.

## 3. Results

### 3.1. Enrichment Analysis of GO Functions and KEGG Pathways

**Figure 2:**
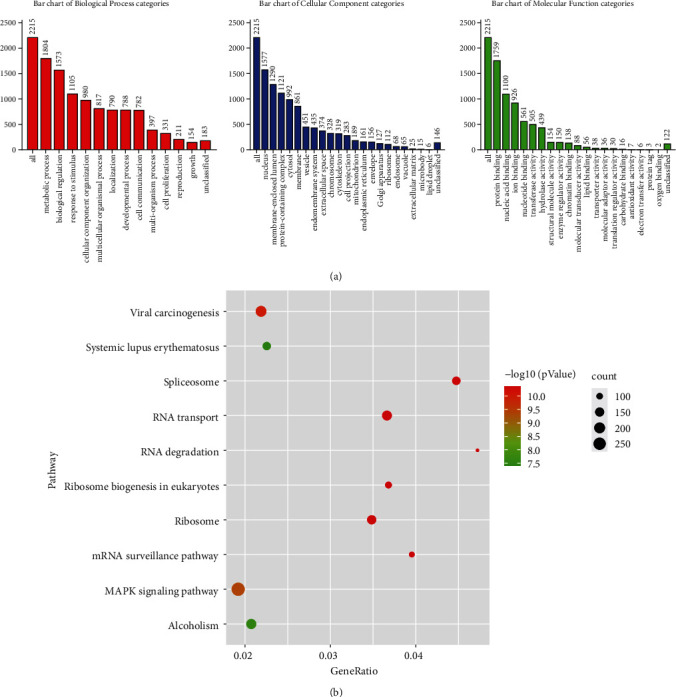
GO functional and KEGG pathway analyses. (a) Summary of the DEG and GO pathway enrichment. Red, blue, and green bars represent the biological process, cellular component, and molecular function categories, respectively. The height of the bar represents the number of DEGs observed in each category. (b) The top 10 pathways of the LLPS genes associated with the OS of HCC patients.

To understand the biological implications of 5207 LLPS genes from the PhaSepDB dataset, we performed GO functions and KEGG pathway analyses. As shown in Figure 2, in the KEGG analysis, we found that these genes were enriched in signalling pathways, including RNA degradation, spliceosome, mRNA surveillance pathway, and ribosome biogenesis in eukaryotes.

### 3.2. DEGs Are Associated with the Overall Survival of HCC Patients

TCGA-LIHC contained 421 samples, including 371 patients with HCC and 50 adjacent normal liver tissues. Of the 371 patients with HCC, enrolled in our study were the 343 patients who had clinical information with corresponding RNA-seq data. We randomly divided the 343 patients into a training dataset (*n* = 240) and a validation dataset (*n* = 103) according to a ratio of 7 to 3. We preliminarily identified 2,364 DEGs at |log_2_FC| > 2 and *P* < 0.05. Then, we performed univariate Cox proportional hazards regression analysis for DEGs to obtain 433 DEGs associated with the OS of HCC patients.

### 3.3. Construction of the Prognostic Signature

**Figure 3:**
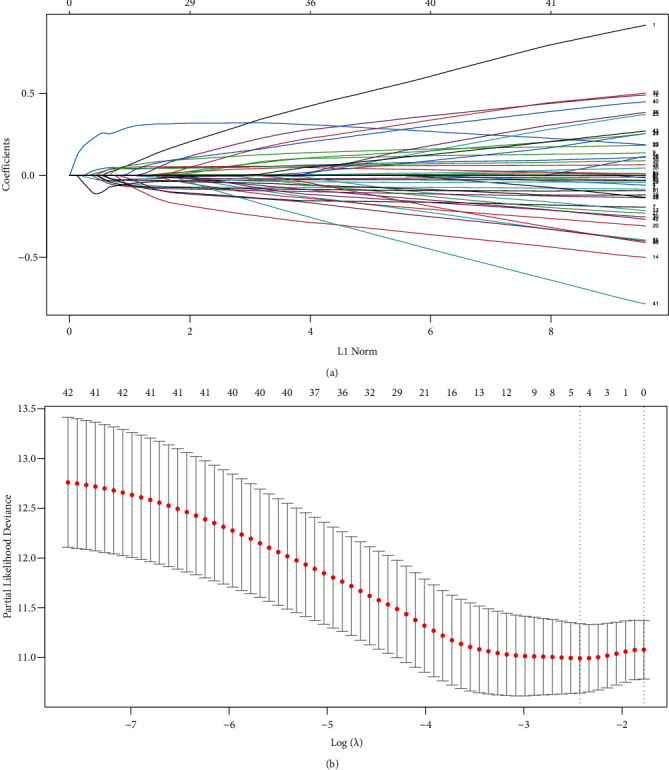
Risk score prognostic signature construction using LASSO Cox penalized regression analysis along with 10-fold cross validation. (a) LASSO coefficient profiles of the LLPS genes associated with the OS of HCC patients. (b) Partial likelihood deviance plotted versus log(lambda). The vertical dotted line indicates the lambda value with the minimum error and the largest lambda value where the deviance is within one standard error (SE) of the minimum.

**Figure 4:**
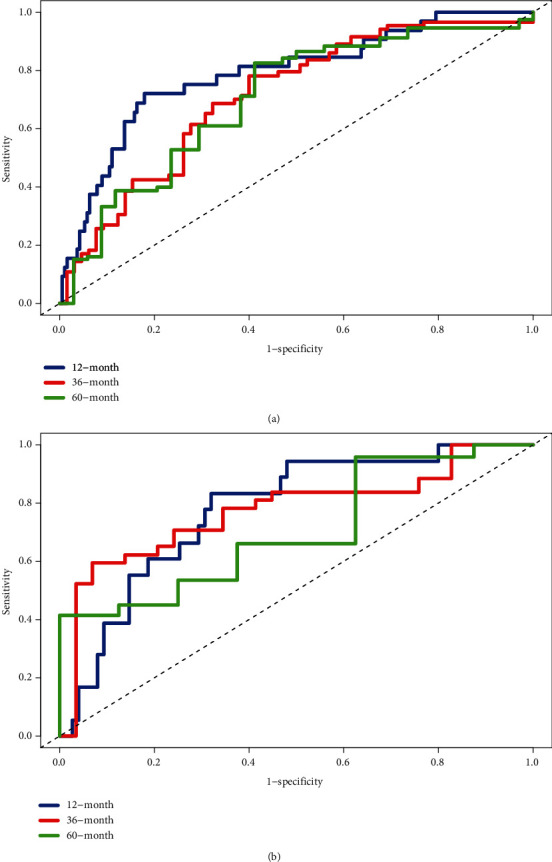
Prediction efficiency of the five-LLPS gene risk score signature evaluated using ROC curves. (a) The ROC curves are shown for the risk score signature in the training dataset. (b) The ROC curves are shown for the risk score signature in the validation dataset.

**Figure 5:**
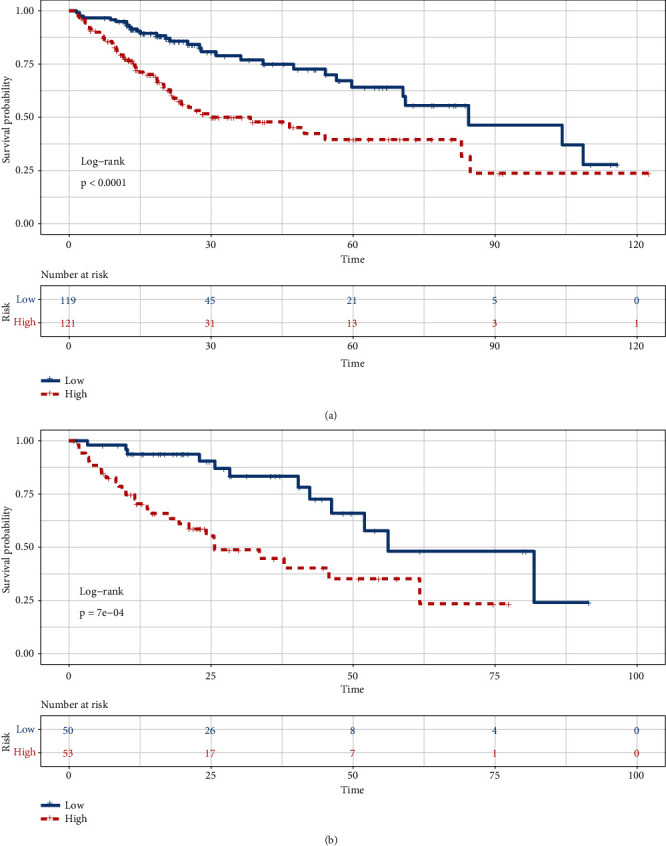
The prognostic role of the five-LLPS gene risk score signature in HCC. (a) The OS of patients in the five-LLPS gene risk score signature low- and high-risk groups in the training dataset. (b) The OS of patients in the five-LLPS gene risk score signature low- and high-risk groups in the validation dataset.

There were 43 genes in the overlapping DEGs associated with the OS of HCC patients and LLPS genes from the PhaSepDB dataset. We constructed a five-LLPS gene (*BMX*, *FYN*, *KPNA2*, *PFKFB4*, and *SPP1*)-based risk score prognostic signature using LASSO Cox penalized regression analysis in the training dataset (Figure 3). The risk score = −0.01833 × expression of *BMX* −0.10197 × expression of *FYN* + 0.22152 × expression of *KPNA2*+0.02634 × expression of *PFKFB4* + 0.00718 × expression of *SPP1*. We performed time-dependent ROC curve analysis for the 12-, 36-, and 60-month survival predictions, and the area under the curve (AUCs) for 12-, 36-, and 60-month OS were 0.789, 0.714, and 0.704, respectively (Figure 4(a)). In addition, according to the results in Figure 5(a), the survival curve revealed that the high-risk group exhibited a worse prognosis than the low-risk group (*P* < 0.001).

### 3.4. The Validation of the Prognostic Signature

We used a dataset from the TCGA-LIHC dataset including 103 patients with complete OS information to evaluate the robustness and effectiveness of the risk score prognostic signature. We also performed time-dependent ROC curve analysis for the 12-, 36-, and 60-month survival predictions. The AUCs for 12-, 36-, and 60-month OS were 0.777, 0.773, and 0.705, respectively (Figure 4(b)). In addition, according to the results in Figure 5(b), the survival curve revealed that the high-risk group exhibited a worse prognosis than the low-risk group (*P* < 0.001). In summary, these results suggested a moderate sensitivity and specificity of the risk score prognostic signature.

### 3.5. Clinical Samples Validation Using Experiments

**Figure 6:**
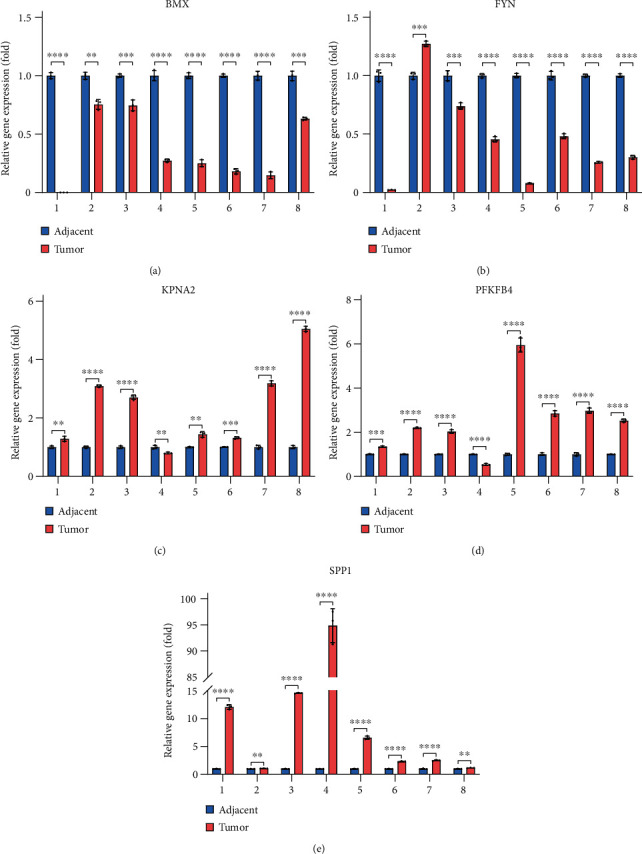
qRT-PCR of eight pairs of tumour tissues and adjacent normal tissues from patients with HCC. (a) Relative gene expression of *BMX*. (b) Relative gene expression of *FYN.* (c) Relative gene expression of *KPNA2*; (d) Relative gene expression of *PFKFB4.* (e) Relative gene expression of *SPP1.* ∗*P* < 0.05; ∗∗*P* < 0.01; ∗∗∗*P* < 0.001; and ∗∗∗∗*P* < 0.0001.

Subsequently, we experimentally validated the genes in the risk score prognostic signature by using eight pairs of HCC clinical tissue samples. qRT-PCR results showed that the expression levels of *BMX* and *FYN* in HCC tumour tissues were significantly lower than those in adjacent normal tissues (Figures 6(a) and 6(b)). Moreover, the expression levels of *KPNA2*, *PFKFB4*, and *SPP1* in HCC tumour tissues were significantly higher than those in adjacent normal tissues (Figures 6(c), 6(d), and 6(e)). These experimental results are consistent with the content of the risk score prognostic signature we constructed. These results indicate that the prognostic signature we constructed has credibility and these genes in the prognostic signature may have corresponding biological functions.

## 4. Discussion

As a new biological mechanism, the study of LLPS can be traced back to 2009 [22]. Previous studies have suggested that LLPS plays an important role in the emergence and development of cancer, including HCC [23, 24]. For example, LLPS of RI***α***, a PKA regulatory subunit, controls cAMP compartmentation as well as oncogenic signalling. The loss of RI*α* LLPS in cells promotes cell proliferation and induces cell transformation in liver cancer [25]. Additionally, it is suggested that biomolecular condensates formed by LLPS may serve as novel therapeutic approach for cancer, since recent evidence showed that these biomolecular condensates influence the pharmacodynamic behaviour of small-molecule drugs [26]. However, the effect of LLPS on the prognosis of HCC patients is currently poorly known.

Notably, previous studies have mainly focused on life activities within or between organelles to predict the prognosis of HCC patients. It seems that the influence of membraneless intracellular biomolecular condensates on cell life activities and patient prognosis has been ignored. To further investigate the prognostic role of the special mechanism in HCC patients and identify relevant targeted therapeutic sites, in this study, we constructed a five-LLPS gene risk score prognostic signature of HCC patients using the TCGA-LIHC dataset and PhaSepDB dataset to discover the effect of LLPS genes on the prognosis of HCC patients.

The result of KEGG analysis showed that these LLPS genes were enriched in signalling pathways, such as RNA degradation, spliceosome, mRNA surveillance pathway, and ribosome biogenesis in eukaryotes. A previous study showed RNA degradation pathway plays an important role in HCC pathogenesis [27]. Another research also demonstrated that genes in spliceosome pathway are up-regulated in HCC by bioinformatic analysis [28]. Our finding suggests that the five-LLPS gene risk score signature might affect the OS of HCC patients through these pathways.

In the training dataset, we constructed a five-LLPS gene (*BMX*, *FYN*, *KPNA2*, *PFKFB4*, and *SPP1*)-based risk score prognostic signature. *BMX* has been demonstrated to show association with the progression of multiple cancers. Guo demonstrated that *BMX* plays a critical role in the regulation of hepatocyte differentiation by c-Fos activation, which contributes to poor survival in some cases of HCC [29]. In addition, other studies have demonstrated that *BMX* can promote the progression of prostate cancer [30] and breast cancer [31]. *FYN*, a nonreceptor tyrosine kinase that belongs to the Src family kinases [32], promotes the development and progression of tumours and is closely related to the prognosis of patients with various tumours [33, 34]. Jiang demonstrated that *KPNA2* is associated with early recurrence and poor prognosis in patients with small HCC [35]. Meanwhile, others studies demonstrated that *KPNA2* knockdown reduced the migration and proliferation capacities of HCC cells [36]. *PFKFB4* was shown to be associated with a poor prognosis in many cancers, such as glioblastoma [37] and bladder cancer [38]. *SPP1* is concerned not only with the occurrence and development of HCC, but also with its poor survival [39–41]. In addition, *SPP1* could promote the progression of ovarian cancer [42] and cause poor survival outcomes in colorectal cancer [43].

In the validation dataset, the AUCs for 12-, 36-, and 60-month OS were 0.777, 0.773, and 0.705, respectively. In addition, the survival curve revealed that the high-risk group exhibited a worse prognosis than the low-risk group (*P* < 0.01). These above results indicated a moderate sensitivity and specificity of the risk score prognostic signature. In addition, we validated the expression levels of these five genes in tumour tissues and corresponding adjacent normal tissues by qPT-PCR. We found that *BMX* and *FYN* had lower expression levels in HCC tumour tissues, whereas *KPNA2*, *PFKFB4*, and *SPP1* had higher expression levels in HCC tumour tissues. These results reveal meaningful biological functions of these genes in HCC, and suggest that these genes play an important role in the development and progression of HCC. All these validation results indicated that the risk score prognostic signature we constructed had good accuracy and credibility.

We constructed risk score prognostic signature using LLPS genes, different from the previous prognostic signatures. To our knowledge, this is the first time that LLPS genes have been used to construct the prognostic signature of HCC patients. Our prognostic signature suggests the potential of targeting LLPS therapeutically for cancer intervention. Meanwhile, with increasing studies on the mechanism of LLPS, the effect of LLPS genes on the prognosis of HCC patients will be more clearly understood.

In a word, our study constructed a novel five-LLPS gene risk score signature for HCC prognosis prediction based on the TAGA-LIHC dataset and PhaSepDB dataset. Our signature might reflect the effect of LLPS genes on the prognosis of HCC patients and suggest the possibility that LLPS genes may serve as a new therapeutic target for cancer intervention.

There are some limitations in our study. First, our study is a retrospective study, and its conclusions are not as powerful as those of prospective studies. Additionally, the sample size of the training dataset and validation dataset in this study was small, which may have led to some unavoidable deviations. Finally, we only analysed the LLPS genes from the PhaSepDB dataset without other LLPS gene datasets in our study, which might contribute to our conclusions not being sufficient. To make the conclusions more reliable, it is necessary to include more LLPS gene datasets for further analysis.

## Figures and Tables

**Table 1:** Information of patients included in the study.

Patient ID	Histological type	Gender	Age (years)	Grade
1	Hepatocellular carcinoma	Male	59	G3
2	Hepatocellular carcinoma	Male	62	G2
3	Hepatocellular carcinoma	Male	54	G2
4	Hepatocellular carcinoma	Female	66	G2
5	Hepatocellular carcinoma	Male	40	G1
6	Hepatocellular carcinoma	Male	83	G2
7	Hepatocellular carcinoma	Female	74	G1
8	Hepatocellular carcinoma	Male	44	G2

**Table 2:** Primers used for qRT-PCR.

Genes	Forward primer (5′ to 3′)	Reverse primer (5′ to 3′)
*GAPDH*	5′-CACCAGGGCTGCTTTTAACTCTG-3′	5′-GATTTTGGAGGGATCTCGCTCCTG-3′
*BMX*	5′-CCCAGACAGAGTGCTGAAGA-3′	5′-TTGAAGATGGTGGCTGGGAG-3′
*FYN*	5′-ACGAGAGGAGGAACAGGAGT-3′	5′-CCCACCAATCTCCTTCCGAG-3′
*KPNA2*	5′-AATCTGCTTGGGCACTCACT-3′	5′-AACACTGAGCCATCACCTGC-3′
*PFKFB4*	5′-CCTACCTCAAGTGTCCGCTG-3′	5′-GAGATGTCCACGTTCTGAGGC-3′
*SPP1*	5′-ACCTGACATCCAGTACCCTGA-3′	5′-ACGGCTGTCCCAATCAGAAG-3′

## Data Availability

Data were downloaded from the TCGA and PhaSepDB website.

## Abbreviations

HCC: Hepatocellular carcinoma
LLPS: Liquid–liquid phase separation
TCGA: The Cancer Genome Atlas
DEGs: Differentially expressed genes
OS: Overall survival
TCGA-LIHC: The Cancer Genome Atlas-liver hepatocellular carcinoma
GO: Gene ontology
KEGG: Kyoto Encyclopedia of Genes and Genomes
LASSO: Least absolute shrinkage and selection operator
ROC: Receiver operating characteristic
qRT-PCR: Quantitative real-time PCR
AUC: Area under the curve.
